# Unexpectedly low nitrogen acquisition and absence of root architecture adaptation to nitrate supply in a *Medicago truncatula* highly branched root mutant

**DOI:** 10.1093/jxb/eru124

**Published:** 2014-04-04

**Authors:** Virginie Bourion, Chantal Martin, Henri de Larambergue, Françoise Jacquin, Grégoire Aubert, Marie-Laure Martin-Magniette, Sandrine Balzergue, Geoffroy Lescure, Sylvie Citerne, Marc Lepetit, Nathalie Munier-Jolain, Christophe Salon, Gérard Duc

**Affiliations:** ^1^INRA, UMR1347 Agroécologie, BP 86510, F-21065 Dijon, France; ^2^INRA, UMR518 MIA, F-75231 Paris, France; ^3^AgroParisTech, UMR MIA, F-75231 Paris, France; ^4^INRA, UMR1165 URGV, F-91057 Evry, France; ^5^UEVE, UMR URGV, F-91057 Evry, France; ^6^CNRS, ERL8196 UMR URGV, F-91057 Evry, France; ^7^Institut Jean-Pierre Bourgin, UMR1318 INRA/AgroParisTech, F-78026 Versailles, France; ^8^USC1342 INRA, UMR113 IRD-CIRAD-SupAgro-UM2, Symbioses Tropicales et Méditerranéennes, Campus de Baillarguet, TA A-82/J, F-34398 Montpellier Cedex 5, France

**Keywords:** Amino acids, highly branched root mutant, *Medicago truncatula*, nitrogen acquisition, nitrogen limitation, phenylpropanoid, root architecture.

## Abstract

To complement N_2_ fixation through symbiosis, legumes can efficiently acquire soil mineral N through adapted root architecture. However, root architecture adaptation to mineral N availability has been little studied in legumes. Therefore, this study investigated the effect of nitrate availability on root architecture in *Medicago truncatula* and assessed the N-uptake potential of a new highly branched root mutant, TR185. The effects of varying nitrate supply on both root architecture and N uptake were characterized in the mutant and in the wild type. Surprisingly, the root architecture of the mutant was not modified by variation in nitrate supply. Moreover, despite its highly branched root architecture, TR185 had a permanently N-starved phenotype. A transcriptome analysis was performed to identify genes differentially expressed between the two genotypes. This analysis revealed differential responses related to the nitrate acquisition pathway and confirmed that N starvation occurred in TR185. Changes in amino acid content and expression of genes involved in the phenylpropanoid pathway were associated with differences in root architecture between the mutant and the wild type.

## Summary

Physiological and developmental analyses provide evidence that the highly branched root architecture of a mutant results from systemic regulation by its nitrogen status, possibly involving glutamine or asparagine signals.

## Introduction

Nitrogen is one of the most limiting resources for plant growth. Legumes have a natural ability to use atmospheric N_2_ as the main N source, via symbiosis in nodules with *Rhizobiaceae* spp. However, N nutrition can still limit yield and seed quality in legumes, especially under abiotic or biotic stress conditions. In these conditions, the fixation of N_2_ is impacted and cannot totally fulfil N demand (Salon *et al.*, 2001), and the poorly developed root systems of N_2_-fixing legumes are unable to explore a large soil volume (Bourion *et al.*, 2007). In this context, the genetic improvement of root system development is a target for increasing legume yield performance.

Up to now, the molecular determinants of root development in legumes have been little characterized. The naturally occurring genetic variability of root development in legumes has been investigated (Kraft and Boge, 2001; McPhee, 2005; Bourion *et al.*, 2010), but few genes involved in root development have been characterized (Yendrek *et al.*, 2010; Jin *et al.*, 2012). A complex tuning of root versus nodule development seems to operate in legumes, as mutants impaired in the autoregulation of nodulation display shorter root length or enhanced lateral root (LR) number (Wopereis *et al.*, 2000; Krusell *et al.*, 2002; Schnabel *et al.*, 2005; Schnabel *et al.*, 2011; Jin *et al.*, 2012). Hormones have been shown to be involved in their common molecular pathways; particularly auxin (de Billy *et al.*, 2001; Jin *et al.*, 2012), cytokinin (Gonzalez-Rizzo *et al.*, 2006; Frugier *et al.*, 2008; Plet *et al.*, 2011), and abscisic acid (Bright *et al.*, 2005; Liang *et al.*, 2007; Yendrek *et al.*, 2010).

The paramount importance of hormones in the regulation of root growth and development has been thoroughly investigated in *Arabidopsis* (Peret *et al.*, 2009). Auxin delivery, which promotes LR initiation, is regulated by the auxin-influx carrier AUX1 and auxin-efflux transporters PINs and PGP/MDR (Muday and DeLong, 2001; Marchant *et al.*, 2002). Auxin transport remains necessary for root elongation (Wu *et al.*, 2007). Interacting effects of auxin and cytokinin disrupt LR initiation by interfering with PIN gene expression and the associated auxin-gradient formation (Laplaze *et al.*, 2007). Cytokinin has been shown to reduce the root elongation rate through an ethylene-induced production (Benkova and Hejatko, 2009; Ruzicka *et al.*, 2009), whereas gibberellin antagonizes the negative effects of ethylene on root growth (Fu and Harberd, 2003; Ubeda-Tomas *et al.*, 2008).

In addition, root growth and development are known in *Arabidopsis* to be modulated by external NO_3_^–^ availability. The localized stimulatory effect of external nitrate on LR elongation has been shown to involve both ANR1 and NRT1.1, which act together as a NO_3_^–^ sensor, promoting auxin transport (Zhang and Forde, 1998; Remans *et al.*, 2006a; Krouk *et al.*, 2010; Gojon *et al.*, 2011). Evidence of roles for cytokinin and abscisic acid in the root architectural response to nitrate have been presented (Walch-Liu *et al.*, 2006a; Kiba *et al.*, 2011; Ruffel *et al.*, 2011). A systemic regulation of the root architecture by the plant N status has been described (Zhang *et al.*, 1999; Remans *et al.*, 2006b), involving feedback repression of root development by products of N assimilation (Walch-Liu *et al.*, 2006b; Gifford *et al.*, 2008). The modulation of the root system architecture in response to N supply is also known to depend on the plant C allocation within the root system (Brun *et al.*, 2010), and LR initiation level has been shown to be related to the C:N ratio (Zhang and Forde, 2000; Malamy and Ryan, 2001; Malamy, 2005). Transcriptomic analyses have confirmed that many genes involved in N assimilation or C primary metabolism are responsive to variation of nitrate supply (Wang *et al.*, 2003; Scheible *et al.*, 2004; Bi *et al.*, 2007). Transcriptomic studies of legumes subjected to variation in nitrate supply are consistent with those obtained in *Arabidopsis* (Ruffel *et al.*, 2008; Omrane *et al.*, 2009).

This study describes a new highly branched root *Medicago truncatula* mutant and showed its unexpectedly low nitrogen acquisition and absence of root architecture adaptation to nitrate supply.

## Materials and methods

### Plant material

*M. truncatula* cv. Jemalong J5 was used as the wild-type reference (WT) and for backcrosses of the mutant TR185. The mutant was selected after gamma-ray mutagenesis on J5 (Sagan *et al.*, 1995) and displayed a phenotype with highly branched roots and few small nodules (Salon *et al.*, 2009). The mutation was stable over four generations of selfing. Genetic analyses revealed that the highly branched root architecture of TR185 is determined by a single recessive mutation (Supplementary Table S1 available at *JXB* online).

### Plant growth conditions

Scarified seeds of both genotypes were surface sterilized for 7min with 3% sodium hypochlorite and rinsed seven times with sterile water. Seeds were then placed on sterilized plastic boxes filled with 1 l of 4% (w/v) Kalys agar HP 696 gel. Boxes were left in the dark for 4 days of cold treatment at 4 °C followed by 4 days of germination at 20 °C. Germinated seeds were transferred to hydroponic culture tanks filled with an aerated nutrient solution. The basal nutrient solution (Ruffel *et al.*, 2008) was supplemented with 1mM KNO_3_ (LN; low nitrate) or 10mM KNO_3_ (HN; high nitrate) as the N source. *Rhizobium* inoculation was performed neither in LN nor HN condition. The two nitrate levels were determined on the basis of previous studies of nitrogen nutrition on *M. truncatula* (Moreau *et al.*, 2008): for non-nodulating plants: optimal N nutrition was achieved with 10mM nitrate supply, whereas the N-nutrition index represented only 35% of the optimum at 0.625–1.25mM nitrate supply. Both nutrient solutions had an initial pH of 6.6 and were renewed every week. Measurements in the hydroponic culture tanks before renewing the solution indicated a slight increase of the pH to 7.2 after 4 weeks, irrespective of N supply. Plants were grown in a growth chamber under a 16/8 light/dark cycle (24/19 °C), mean photosynthetically active radiation 200 μmol photons m^–2^ s^–1^, and 70% relative humidity. Each tank contained six WT and six TR185 plants. On one shelf of the growth chamber, nutrient solution in the tanks was supplemented by 1mM KNO_3_ (LN); on the other shelf, the concentration of KNO_3_ in the solution was 10mM (HN). Three successive experiments in the growth chamber were performed on the two different genotypes. Each experiment constituted a biological replicate. In each experiment, plants were collected at five successive dates from 7 to 28 days after the transfer of germinated seeds into the tanks.

### Plant measurements, ecophysiological modelling, and grafting

At each of the five dates, six plants of each genotype were collected both in one LN and one HN tank. Length of the primary root (PRL) was measured. The first-to-third-order lateral roots were counted, allowing the calculation of total lateral root number (LRN). No nodule was found in any root observed. Then, the shoot and root systems were carefully spread separately onto transparent sheets and scanned as digital images with an A3 colour scanner (Epson, Tokyo, Japan). Total leaf area (LeafA), total root length (TRL), and total root surface area per plant were further determined by image analysis using WinRhizo software (Regent Instruments, Quebec, Canada). Mean lateral root length (LRL) was then calculated as (TRL – PRL)/LRN. Roots and shoots were ovendried separately at 80 °C for 48h for shoot, root, and total dry weight determination (SDW, RDW, and TDW). Shoot and root N concentrations of ground dried tissues (%ShootN, %RootN) were estimated following the Dumas’ method, and the total N accumulation in the plant (TotN) calculated. Then, three integrative variables, characterizing the relationship between the four state variables LeafA, TDW, RDW, and TotN were calculated (Moreau *et al.*, 2012) to represent efficiency of C or N acquisition. The LeafA is considered as the C source, which is distributed to roots according to root-to-total dry weight ratio (RDW/TDW). The RDW, or more precisely the root surface area, pilots the entrance of N into the plant, according to N-uptake rate (NUR). The TotN accumulated into the plant allows the elaboration of the LeafA, according to efficiency of N conversion into leaf area (NLA).

Grafting was performed as described in the ‘cuttings and grafts’ chapter of the *Medicago* handbook (http://www.noble.org/medicagohandbook/). Grafts were initially generated *in vitro*, and after 3 weeks were potted in attapulgite:clay balls (1:1) in the greenhouse for an additional 7 weeks. Three plants per combination were then carefully spread onto transparent sheets and scanned as digital images, and LeafA and TRL determined by image analysis. The number of first lateral roots per length of primary root was also determined for each plant.

For graft experiment and each sampling date, means and SE values were calculated for all variables and ANOVA were performed using XLSTAT software (version 2010.6.03, http://www.xlstat.com). Means were classified using the least significant difference (LSD) range test at the 0.05 probability level.

### Metabolic analyses

#### Amino acid content

The levels of the 20 standard amino acids synthesized by plants were measured in TR185 and WT plants. Lyophilized samples (100mg) were extracted in a three-step ethanol/water procedure, as described by Loudet *et al.* (2003). Using the method described by Ikram *et al.* (2012), ninhydrin colour reagent was added to the extract and absorbance read at 570nm on a spectrophotometer. This result was used to calculate the amino acid content in μmol (g FW)^–1^.

#### Lignin content

Lignin content in roots was determined using the acetyl bromide method adapted from Fukushima and Hatfield (2001). To prepare the root cell wall, 100mg dried and ground root samples were extracted sequentially with stirring with water, ethanol, and acetone. An acetyl bromide/acetic acid solution (1:3, v/v) was added to about 5mg dried extract. Lignins were solubilized whereas polysaccharides were hydrolysed. After the reaction, the excess of acetyl bromide and polybromide ions were destroyed by adding water and hydroxylamine chlorhydrate. Lignin content was calculated from absorbance readings at 280nm, and expressed as mg (g root DW)^–1^.

### Transcriptomic analyses

#### RNA extraction and Affymetrix GeneChip

Total RNA was extracted from roots using the Plant RNeasy Mini Kit with on-column DNAse digestion (Qiagen). All RNA samples were checked for their integrity on the Agilent 2100 Bioanalyzer (Agilent Technologies, Waldbronn, Germany) according to the manufacturer’s instructions. For microarray analyses, 2 μg total RNA were transcribed as described in Rey *et al.* (2013). The labelled cDNA produced was used to hybridize Affymetrix GeneChip *Medicago* genome arrays at INRA-URGV (Evry, France). The raw CEL files were imported in R software for data analysis. All raw and normalized data are available through the CATdb database (Gagnot *et al.*, 2008; project ‘AFFY_root_dvt_Nitrogen_*Medicago*’), and from the Gene Expression Omnibus (GEO) repository at the National Center for Biotechnology Information (NCBI) (Barrett *et al.*, 2007; accession number GSE18318).

#### Statistical analysis of microarray data

The data were normalized with the GC RMA algorithm (Irizarry *et al.*, 2003) available in the Bioconductor package (Gentleman and Carey, 2002). We performed a two-way ANOVA on the normalized expression signals, which was modelled as follows: *Y*_ijk_ = μ + *G*_i_ + *N*_j_ + GN_ij_ + *E*_ijk_, where *Y* is the normalized expression signal of a transcript for genotype i at nitrate supply j in replicate k, μ the global mean, *G*_i_ the genotypic effect, *N*_j_ the nitrate effect, GN_ij_ the genotype×nitrate interaction effect, and *E*_ijk_ are normally distributed zero-mean random errors. Due to the limited number of observations, the degree of freedom was too weak to perform tests based on the specific residual variance of each transcript. Thus, a global residual variance was calculated after the removal of the transcripts displaying extreme variation. Three contrasts were considered to classify genes as responsive to the genotype effect independently of nitrate supply (G), responsive to nitrate supply across both genotypes (N), or not responsive to nitrate supply in the same way in both genotypes (G×N interaction). For each contrast, the test statistic was calculated from the global variance, and *P*-values were adjusted by the Bonferroni method, which controls the family-wise error rate (Ge *et al.*, 2003). For a given contrast, a gene is declared differentially expressed if its adjusted *P*-value is lower than 0.05. A functional classification of the differentially expressed genes was visualized using MapMan version 3.5.0 (http://mapman.gabipd.org/web/guest/mapman; Thimm *et al.*, 2004; Tellstrom *et al.*, 2007).

#### Quantitative reverse-transcription PCR

A set of 18 genes identified as differentially expressed in roots was chosen for validation of Affymetrix genome arrays by quantitative reverse-transcription PCR (Supplementary Fig. S1 available at *JXB* online). Primer sequences are available in Supplementary Table S2 (available at *JXB* online). For each sample, 1 μg total RNA was treated with RQ1 DNAse (Promega) and reverse transcription was carried out using the IScript cDNA synthesis kit (BIO-RAD). Reactions were performed on a LC480 apparatus (Roche) using the GoTaq qPCR Mastermix (Promega). Three technical replicates were performed for each one of the three independent biological replicates. Relative expression levels were calculated according to the relative standard curve method (ΔCT) using Elongation Factor 1 and Ubiquitin genes as reference genes.

## Results

### Root architecture and N uptake

#### The highly branched root architecture of TR185 is associated with depressed growth irrespective of nitrate supply and is shoot determined

**Fig. 1.**
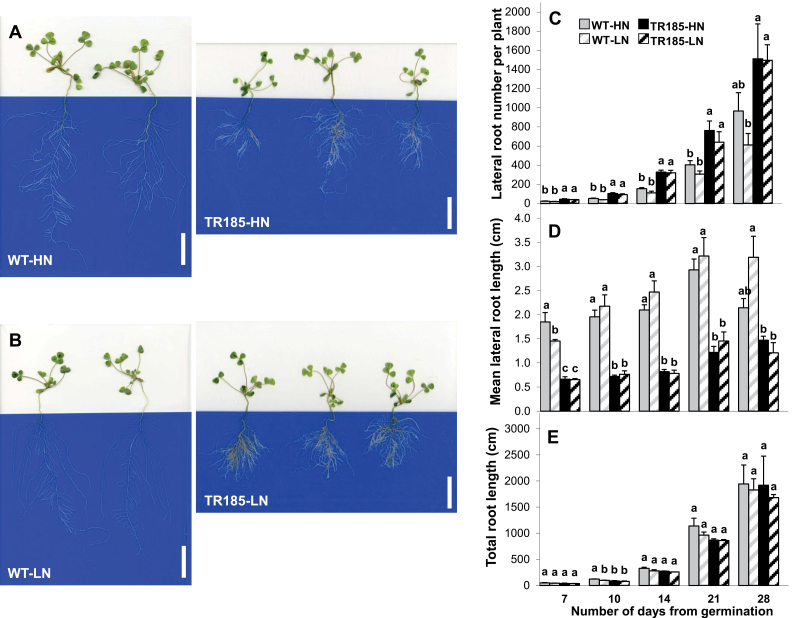
Highly branched root architecture of the mutant TR185 irrespective of nitrate supply. (A and B) Representative examples of wild-type (WT) and TR185 plants grown for 14 days in a growth chamber on hydroponic culture tanks filled with nutrient solutions with high (A; 10mM, HN) or low (B; 1mM, LN) nitrate supply. Bars, 5cm. (C–E) Quantification of root architecture from 7 to 28 days after germination: lateral root number per plant (C), lateral root length (D), and total root length per plant (E). Data are mean±SE from three biological replicates of six plants each. Different letters above the columns indicate significant difference based on multiple comparisons (*P*<0.05, LSD test). The decrease in lateral root length at 28 days after germination in WT plants grown in HN has no biological significance (this figure is available in colour at *JXB* online).

The root architecture of TR185 was highly branched under both high and low nitrate supply (Fig. 1). This resulted in a significantly higher LRN and significantly lower mean LRL compared with WT, throughout the growth period and irrespective of nitrate supply (Fig. 1C and D). Despite this, TR185 had a similar total root length as WT plants (Fig. 1E). In WT plants, LRN decreased slightly with decreasing nitrate supply (Fig. 1C). An effect of decreased nitrate supply was also observed on the LRL of the WT, with a transient significant decrease at the 7-day stage followed by an increase at the later stages (Fig. 1D).

**Fig. 2.**
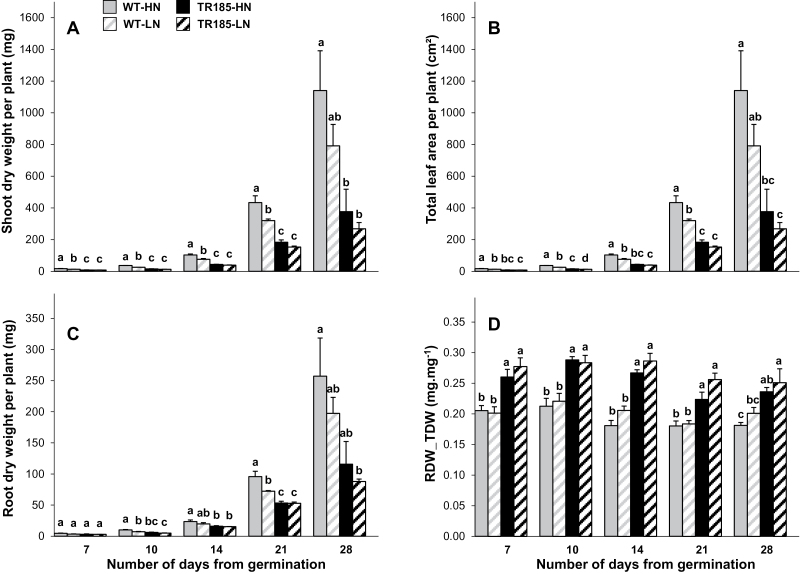
Dry weight and leaf area of wild-type (WT) and mutant (TR185) plants under high (10mM, HN) or low (1mM, LN) nitrate supply from 7 to 28 days after germination. (A) Shoot dry weight. (B) Total leaf area. (C) Root dry weight per plant. (D) Root-to-total dry weight ratio. Data are mean±SE from three biological replicates of six plants each. Different letters above the columns indicate significant difference based on multiple comparisons (*P*<0.05, LSD test).

TR185 displayed significantly lower SDW, RDW, and leaf area (LeafA) than WT as early as the 7- or 10-day stage (Fig. 2A–C). These differences were associated throughout the growth period with a higher root-to-total dry weight ratio in TR185 compared with WT (Fig. 2D). Both RDW and SDW of the WT were reduced with LN supply, whereas no significant decrease was observed in TR185.

**Fig. 3.**
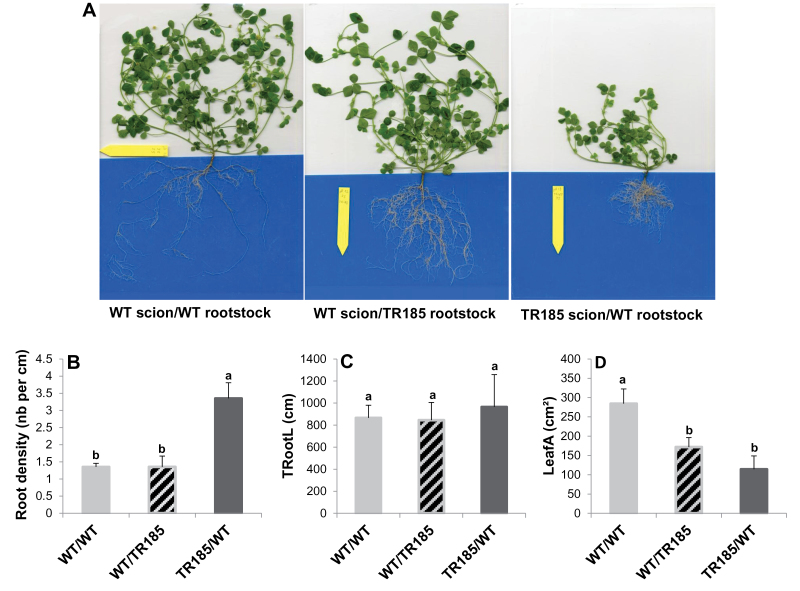
Grafting experiments. (A) Representative 10-week old plants. (B) Lateral roots per length of primary root (root density). (C) Total root length (TRootL). (D) Total leaf area (LeafA) of 10-week old plants. Data are mean±SE from three plants (this figure is available in colour at *JXB* online).

To determine whether the highly branched root architecture of TR185 was shoot or root determined, this study performed grafting experiments (Fig. 3). Analysis of roots in the different grafting combinations revealed that the root architecture phenotype was graft transmissible from shoots (Fig. 3A–C). The shoot leaf area was also most reduced with TR185 as the scion (Fig. 3D).

#### Compared with WT, TR185 has reduced shoot N concentration and N-uptake efficiency

**Fig. 4.**
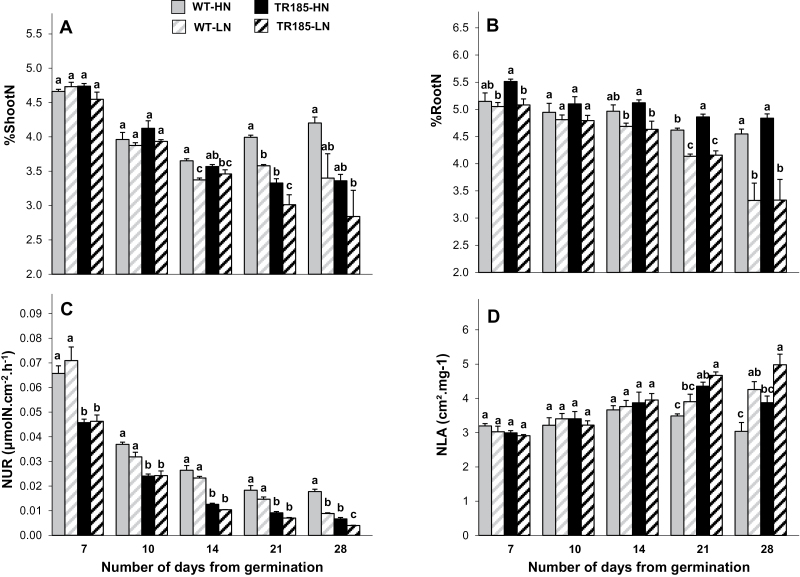
N concentration and efficiency of nitrogen accumulation into wild-type (WT) and mutant (TR185) plants under high (10mM, HN) or low (1mM, LN) nitrate supply from 7 to 28 days after germination. (A and B) Shoot N concentration (A; %ShootN) and root N concentration (B; %RootN) estimated following the Dumas method. (C and D) N-uptake rate (C; NUR) and efficiency of N conversion into leaf area (D; NLA). Data are mean±SE from three biological replicates of six plants each. Different letters above the columns indicate significant difference based on multiple comparisons (*P*<0.05, LSD test).

Up to the 14-day stage, %ShootN decreased continuously for both genotypes without significant difference between them (Fig. 4A). From the 21-day stage onwards, %ShootN became significantly lower in TR185 than in WT, and the depressing effect of LN was significant for both genotypes. TR185 has lower %Shoot N than WT even when normalized to SDW measures (Supplementary Fig. S2 available at *JXB* online). Few differences in root N concentration (%RootN) were significant between the two genotypes (Fig. 4B). For both genotypes, %RootN decreased throughout the study period, especially under LN supply.

The N-uptake rate was significantly lower in TR185 than in WT, as soon as the 7-day stage and under both nitrate conditions (Fig. 4C). For both genotypes, NUR decreased throughout the growth period and, at the 28-day stage, was significantly lower under LN supply. Concerning the amount of leaf area produced per N acquired by the roots (NLA), significantly higher values were observed for TR185 from the 21-day stage onwards (Fig. 4D). From that stage, both genotypes had higher NLA values under LN than HN supply.

#### TR185 has lower ASN and GLN contents than WT

**Fig. 5.**
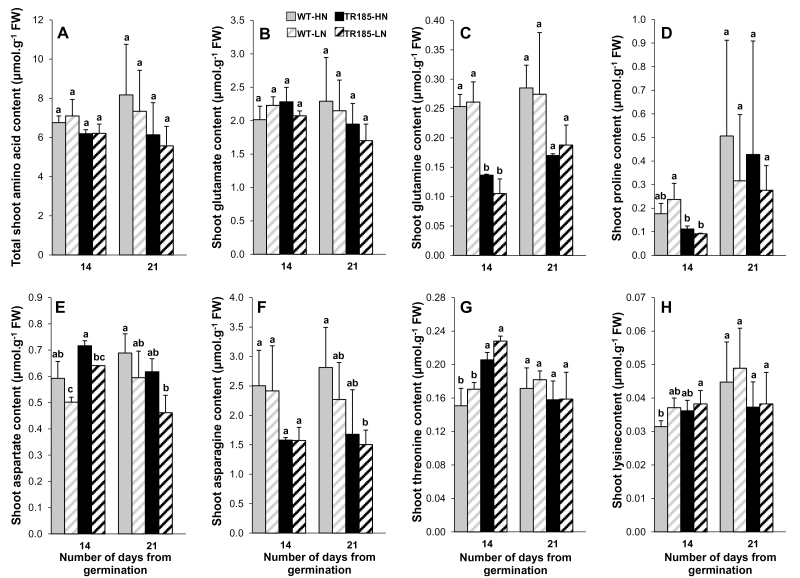
Total amino acid content and contents of seven amino acids in wild-type (WT) and mutant (TR185) shoots, under high (10mM, HN) or low (1mM, LN) nitrate supply at 14 and 21 days after germination. Data are mean±SE from three biological replicates of six plants each. Different letters above the columns indicate significant difference based on multiple comparisons (*P*<0.05, LSD test).

The levels of the 20 standard amino acids synthesized by plants were measured at 14 and 21 days. No differences in total free amino acid or glutamate (GLU) content in shoots were observed between TR185 and the WT or between LN and HN supply at the 14-day stage, whereas differences none significant but similar to that observed in %ShootN appeared at the 21-day stage (Fig. 5A, B). Six other amino acids did not show any significant variations at the two stages considered (Supplementary Fig. S3B–E, H–J available at *JXB* online). In contrast, significant differences between the two genotypes in the contents of eight amino acids were observed as soon as the 14-day stage, with lower values for glutamine (GLN), proline (PRO), asparagine (ASN), and alanine (ALA) (Fig. 5C,D,F; Supplementary Fig. S3A available at *JXB* online) and higher values for threonine (THR), lysine (LYS), valine (VAL), and serine (SER), in TR185 compared with WT (Fig. 5G; Supplementary Fig. S3B,F available at *JXB* online). For three amino acids, aspartate (ASP), SER, and cysteine (CYS), a significant effect of N supply was observed (Fig. 5E; Supplementary Fig. S3F, G available at *JXB* online).

**Fig. 6.**
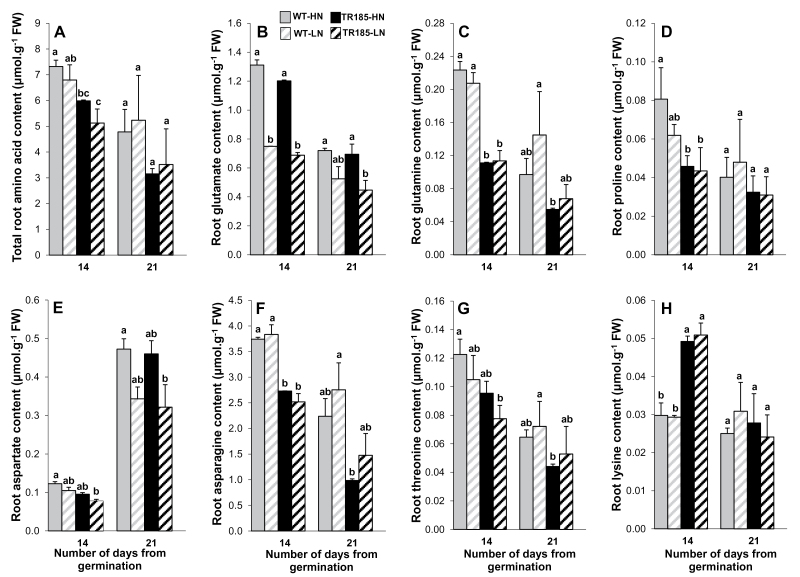
Total amino acid content and contents of seven amino acids in wild-type (WT) and mutant (TR185) roots, under high (10mM. [HN]) or low (1mM. [LN]) nitrate supply, and at 14 and 21 days after germination. At each date, data are means from three biological replicates of six plants each ± SE. Means followed by different letters are significantly different based on multiple comparisons (LSD test) at p < 0.05.

A lower total free amino acid content was observed in the roots of TR185 compared with WT roots (Fig. 6A). However, no significant differences were observed between TR185 and the WT for their root levels of GLU and ASP, and for both genotypes, the level of these two amino acids was lower under LN than under HN supply (Fig. 6B, E). By contrast, TR185 had a lower level of GLN and ASN, and no significant effect of N supply was observed for these two amino acids (Fig. 6C, F). A genotype effect was also observed for PRO, THR, and LYS, at least at the 14-day stage (Fig. 6D, G, H). Arginine (ARG), histidine (HIS), isoleucine (ILE), SER, phenylalanine (PHE), and all the derivatives of pyruvate (ALA, VAL, LEU) did not show any significant variations (Supplementary Fig. S4 available at *JXB* online).

### Transcriptomic analysis

#### Most genes are differentially expressed in TR185 and WT

Global gene expression profiling of root cells using a microarray analysis was conducted on both the WT and TR185 under HN and LN. The transcriptomic analysis was performed on the 10-day stage, at which the two genotypes were significantly different for most of the traits related to the root architecture or plant growth.

**Fig. 7.**
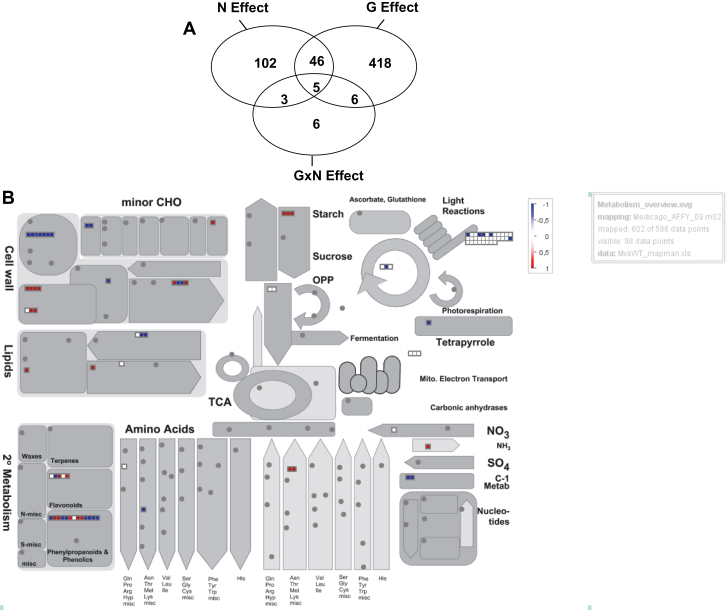
Comparisons of mutant (TR185) and wild-type (WT) transcriptomes. (A) Venn diagram of transcripts identified as differentially expressed in roots in response to genotype effect (G), to nitrate supply across both genotypes (N), or to genotype according to nitrate supply (G×N interaction). (B) Overall picture of gene expression changes in roots between TR185 and WT in the main metabolic pathways. This MapMan representation is based on annotations of *Medicago*_AFFY_09 *M. truncatula* microarrays. Only transcripts significantly differentially expressed are shown. Differential values are expressed in a log_2_ scale. Transcripts differentially expressed by more than the threshold value of 1 are shown in colour; red for upregulated and blue for downregulated in TR185; in both cases with a colour scale representing the intensity of up- or downregulation. Absent transcripts are shown in grey.

Significant hybridization in at least one root sample was found on 26 754 probe sets among the 61 278 tested (Filter based on signal values >4). Of these, 586 were differentially expressed; among them, 475 were differentially expressed in response to genotype effect (G) independently of nitrate supply, 156 in response to nitrate effect (N) across both genotypes, and 20 in response to G×N effect (Fig. 7A). Several of these genes were responsive to either two or three effects in common. Of the 168 transcripts responding to N or G×N effects, 77 were previously identified by Ruffel *et al.* (2008) to be regulated in WT roots in response to either local nitrate starvation (65 transcripts) or to systemic signals related to the plant N status (20 transcripts), with eight in response to both signals (Supplementary Table S3 available at *JXB* online). Fifty-six of the common transcripts previously found to be upregulated in response to local nitrate starvation by Ruffel *et al.* (2008) were significantly upregulated in LN compared with HN. Altogether, these results confirmed that the LN treatment resulted in N limitation.

Using the MapMan software, an overall comparison of the main metabolic pathways highlights differential gene expression between TR185 and WT in N acquisition and amino acid synthesis, cell-wall and lipid metabolism, and phenylpropanoid and flavonoid biosynthetic pathways (Fig. 7B).

#### Most genes involved in N acquisition and assimilation are upregulated in TR185 compared with WT

Among the annotated transcripts differentially expressed in roots between TR185 and WT, 23 are involved in the N acquisition and assimilation pathway (Table 1). One of the most upregulated transcripts in TR185 encodes a putative nitrate transporter of the NRT2 family (Table 1). A transcript encoding a putative NRT1 nitrate transporter and two transcripts encoding putative ammonium transporters of AMT1 and AMT2 family were also more highly expressed in TR185. Concerning the ammonium assimilation, a transcript encoding a glutamate synthase was upregulated in TR185 under HN supply but downregulated under LN supply. A transcript encoding a pyrroline-5-carboxylate synthetase, which is involved in conversion of GLU to PRO, was downregulated, in TR185 compared with WT, under HN supply and upregulated under LN supply. A transcript encoding a glutamate dehydrogenase was more highly expressed in TR185 than in the WT irrespective of nitrate supply. Two transcripts encoding an l-asparagine amidohydrolase involved in ASN degradation were upregulated in TR185 compared with WT, whereas the expression of a transcript encoding a dihidropicolinate synthase involved in LYS synthesis was downregulated.

**Table 1.** Differentially accumulated transcripts between TR185 and wild type, annotated as related to N-acquisition pathwayAverage Affymetrix GeneChip normalized expression values across three biological replicates for the mutant TR185 and the wild type in high and low N conditions. G, N, and G×N indicate transcripts responsive to genotype, nitrate, and genotype×nitrate effect, respectively (adjusted *P*-values <0.05). G* indicates a transcript responsive to G effect only under HN condition. N* indicates a transcript responsive to N effect only for the wild type. *G* and G indicate upregulation and downregulation, respectively, in TR185 compared with the wild type.

Target identifier	Annotation	MHN	MLN	WTHN	WTLN	Effect
Putative nitrate transporters						
Mtr.44342.1.s1_at	NRT2 Nitrate transporter	5.13	4.15	2.76	3.19	G
Mtr.47690.1.s1_s_at	NRT1 Nitrate transporter	7.10	6.97	6.19	5.81	G
Ammonium transporters						
Mtr.1706.1.s1_s_at	AMT1 transporter	7.78	8.54	6.75	7.43	G
Mtr.25576.1.S1_at	AMT2 transporter	7.60	7.11	5.89	5.21	G
Ammonium assimilation						
Mtr.33664.1.s1_at	Ferredoxin-dependent glutamate synthase	4.53	3.60	3.58	4.55	G×N
Mtr.42847.1.s1_at	Pyrroline-5-carboxylate synthetase	8.80	9.58	9.83	8.66	G×N
Mtr.43170.1.s1_s_at	Glutamate deshydrogenase	11.81	12.07	11.04	10.90	G
Mtr.25985.1.s1_at	Putative l-asparagine amidohydrolase	14.05	13.66	12.57	12.76	G
Msa.1503.1.s1_at	Putative l-asparagine amidohydrolase	12.92	12.80	11.40	11.72	G
Mtr.39486.1.s1_at	Dihydrodipicolinate synthase	3.27	4.48	6.19	6.77	G
Putative amino acid transporters						
Mtr.12447.1.s1_at	Proline transporter	9.30	9.68	10.30	10.63	G
Mtr.31521.1.s1_at	Lysine histidine transporter	8.03	8.52	6.77	6.99	G
Mtr.38963.1.s1_at	Amino acid transporter	5.82	6.28	4.74	5.43	G
Putative peptide transporters						
Mtr.7014.1.s1_at	NRT1 peptide transporter	9.21	9.63	8.22	8.62	G
Mtr.48790.1.s1_at	Proton-dependent oligopeptide transporter	5.15	4.42	3.92	3.50	G
Organic acid metabolism						
Mtr.32662.1.s1_at	beta-Amylase	7.24	7.39	6.25	6.30	G
Mtr.13958.1.s1_at	beta-Amylase	6.87	7.13	5.82	5.73	G
Mtr.12555.1.s1_at	Starch phosphorylase	5.15	5.07	3.41	3.97	G
Mtr.23663.1.S1_at	Phosphoenolpyruvate carboxylase kinase	7.45	6.57	5.94	7.53	N* G×N
Sugar transporters						
Mtr.12578.1.s1_at	Malate transporter	10.85	11.33	9.95	10.32	G
Mtr.33536.1.s1_at	Sugar transporter	5.47	4.89	4.10	5.24	G*
Mtr.19796.1.s1_at	Glucose transporter	7.65	8.58	8.97	9.37	G
Msa.905.1.S1_at	Leghaemoglobin MtLb1	6.51	6.64	6.04	4.37	G

A differential expression between TR185 and WT was also observed for genes involved in the synthesis of organic acids which are required for N assimilation. Transcripts involved in starch degradation were upregulated in TR185 compared with WT, irrespective of N supply for those encoding beta-amylase and starch phosphorylase, and under HN supply only for one encoding a phosphoenolpyruvate carboxylase kinase. Differential expressions of transcripts encoding sugar transporters were observed also between TR185 and WT.

#### Genes involved in cell-wall and lipid metabolism are differentially expressed in TR185 and WT

Twenty-four of the annotated transcripts differentially expressed between TR185 and WT were found to be involved in cell-wall modification or lipid metabolism (Fig. 7B; Table 2). Most of the transcripts encoding cell-wall-modifying enzymes, such expansins, pectinesterases, and polygalacturonases, were upregulated in TR185. Other notable changes in TR185 concerned differential expression of transcripts encoding cell-wall polysaccharide synthases, with downregulation of a cellulose synthase and upregulation of a callose synthase. Lastly, most of the transcripts encoding cell-wall arabinogalactan proteins or lipid-binding proteins were downregulated in TR185. Concerning the lipid metabolism, the main changes in expression between TR185 and WT were the downregulation of transcripts related to fatty acid elongation and lipid synthesis and the upregulation of a transcript encoding a lipase.

**Table 2.** Differentially accumulated transcripts between TR185 and wild type, annotated as related to cell-wall modificationAverage Affymetrix GeneChip normalized expression values across three biological replicates for the mutant TR185 and the wild type in high and low N conditions. G and N indicate transcripts responsive to genotype and nitrate effect, respectively (adjusted *P*-values <0.05). *G* and G indicate upregulation and downregulation, respectively, in TR185 compared with the wild type.

Target identifier	Annotation	MHN	MLN	WTHN	WTLN	Effect
Cell-wall-modifying enzymes						
Mtr.9830.1.s1_at	Expansin	8.23	7.88	6.86	7.37	G
Mtr.20107.1.s1_at	Expansin-related protein precursor	8.04	7.73	9.31	8.87	G
Mtr.22752.1.s1_s_at	Expansin	9.64	9.93	8.82	8.74	G
Mtr.4467.1.s1_at	Pectinesterase	11.16	10.88	10.15	9.08	G
Mtr.274.1.s1_at	Pectinesterase	7.54	7.79	6.78	5.86	G
Mtr.7581.1.s1_s_at	Pectinesterase	9.13	8.88	8.36	7.33	G
Mtr.41480.1.s1_at	Polygalacturonase	6.65	7.55	5.86	6.33	G
Mtr.4713.1.s1_at	Lyase	5.39	5.93	4.75	4.51	G
Mtr.39445.1.s1_at	Polygalacturonase	6.93	8.04	8.13	8.79	G, N
Mtr.43680.1.s1_at	Dehydration-induced protein	7.07	8.07	8.41	8.78	G
Cell-wall polysaccharides						
Mtr.28768.1.s1_at	Cellulose synthase	6.46	6.83	7.56	8.00	G
Mtr.17447.1.s1_at	Callose synthase	5.47	5.42	3.69	4.81	G
Cell-wall arabinogalactan proteins						
Mtr.18563.1.s1_at	Fasciclin-like arabinogalactan protein	6.88	7.55	7.98	8.56	G
Mtr.51607.1.s1_at	Fasciclin-like arabinogalactan protein	10.26	11.14	11.47	11.95	G
Mtr.50900.1.s1_at	Fasciclin-like arabinogalactan protein	8.82	9.53	9.79	10.46	G
Mtr.10992.1.s1_at	Fasciclin-like arabinogalactan protein	10.13	10.82	11.17	11.77	G
Mtr.18380.1.s1_at	Fasciclin-like arabinogalactan protein	9.05	9.69	10.05	10.76	G
Mtr.50897.1.s1_at	Fasciclin-like arabinogalactan protein	6.81	7.51	7.95	8.60	G
Mtr.13136.1.s1_at	Fasciclin-like arabinogalactan protein	9.09	9.82	10.19	10.75	G
Mtr.32740.1.S1_at	Lipid-binding protein	10.45	9.68	8.99	9.00	G
Mtr.37476.1.S1_at	Lipid-binding protein	8.35	8.84	9.73	9.39	G
Lipid metabolism						
Mtr.12519.1.s1_at	beta-Ketoacyl-CoA synthase	4.88	5.11	5.82	6.17	G
Mtr.41116.1.s1_at	Acyl carrier protein	3.59	3.66	5.08	4.68	G
Mtr.12518.1.s1_at	Lipase	11.47	11.86	10.55	10.74	G

#### Most genes involved in the phenylpropanoid pathway are upregulated in TR185 compared with WT

Twenty-three of the annotated transcripts differentially expressed between TR185 and WT were found to be involved in the phenylpropanoid and flavonoid pathways (Fig. 7B; Table 3). A large number of transcripts involved in lignin synthesis were repressed in TR185 compared with WT, including those encoding HCT and caffeoyl-CoA 3-*O*-methyltransferase. Conversely, a transcript encoding a chalcone synthase, which is a crucial flavonoid biosynthesis enzyme, and numerous transcripts involved in flavonol glycoside synthesis were upregulated in TR185 compared with WT, independently of nitrate supply. Various transcripts involved in the anthocyanin pathway were also differentially expressed between TR185 and WT; with upregulation of the transcription factor ANL2 and downregulation of an anthocyan-5-aromatic acyltransferase and a gene similar to *tt12*, both irrespective of nitrate supply.

**Table 3.** Differentially accumulated transcripts between TR185 and wild type, annotated as related to phenylpropanoid pathwayAverage Affymetrix GeneChip normalized expression values across three biological replicates for the mutant TR185 and the wild type in high and low N conditions. G, N, and G×N indicate transcripts responsive to genotype, nitrate, and genotype×nitrate effect, respectively (adjusted *P*-values <0.05). *G* and G indicate upregulation and downregulation, respectively, in TR185 compared with the wild type.

Target identifier	Annotation	MHN	MLN	WTHN	WTLN	Effect
Phenylpropanoid pathway						
Mtr.40166.1.s1_s_at	Phenylalanine ammonia-lyase	10.39	11.19	9.46	10.18	G
Mtr.12988.1.s1_at	4-Coumarate-CoA ligase	6.64	6.68	7.54	7.71	G
Lignin pathway						
Mtr.20618.1.s1_s_at	Transferase family protein (HCT)	5.94	7.29	7.58	7.86	G
Mtr.4076.1.s1_at	Caffeoyl-CoA 3-*O*-methyltransferase	5.45	5.80	6.22	6.95	G
Mtr.40942.1.s1_at	Caffeoyl-CoA 3-*O*-methyltransferase	4.26	4.52	5.36	5.39	G
Mtr.10331.1.s1_at	Isoflavone-*O*- methyltransferase	9.49	9.80	10.44	10.79	G
Mtr.51214.1.s1_at	*O*-Methyltransferase	3.21	3.42	3.97	4.75	G
Flavonoid pathway						
Mtr.49423.1.s1_at	Chalcone synthase	5.01	4.81	4.15	3.22	G
Dihydroflavonol pathway						
Mtr.20354.1.S1_at	UDP-Glucose flavonol 3-*O*-glucosyltransferase	7.81	7.03	6.09	5.46	G
Mtr.28721.1.s1_at	UDP-Glucose flavonol 3-*O*-glucosyltransferase	6.46	6.06	5.32	5.25	G
Mtr.44246.1.S1_at	UDP-Glucose flavonol 3-*O*-glucosyltransferase	6.15	5.29	4.89	3.97	G
Mtr.27374.1.S1_at	UDP-Glucosyltransferase	6.82	6.33	4.94	5.39	G
Mtr.37046.1.s1_at	UDP-Glucosyltransferase	8.45	7.81	7.37	6.82	G
Mtr.9669.1.s1_at	Transferase	7.77	7.10	6.61	6.35	G
Mtr.27554.1.s1_at	Transferase	6.94	6.37	5.83	5.23	G
Mtr.29306.1.s1_at	Transferase	8.30	8.92	7.19	7.58	G
Mtr.10626.1.s1_at	Flavonol synthase/flavanone 3-hydroxylase	11.62	11.40	10.88	10.21	G
Mtr.14782.1.s1_at	Flavonoid biosynthetic process DMR6	5.54	5.20	4.17	4.75	G
Anthocyanin pathway						
Mtr.31382.1.s1_at	Dihydroflavonol 4-reductase	7.99	8.72	8.97	7.72	G×N
Mtr.30762.1.s1_at	Anthocyanin 5-aromatic acyltransferase	4.90	5.37	6.31	6.03	G
Mtr.43878.1.s1_at	Anthocyaninless2 transcription factor	7.83	6.97	6.29	5.95	G
Mtr.35206.1.s1_at	Anthocyaninless2 transcription factor	6.41	5.59	4.65	4.34	G
Mtr.2481.1.S1_at	Protein Transparent Testa 12	2.87	3.62	3.34	5.03	G N

#### Genes involved in hormone metabolism and transport are differentially expressed in TR185 and WT

Twenty-three of the annotated transcripts differentially expressed between TR185 and WT were found to be involved in hormone metabolism or transport (Table 4). The transcripts encoding auxin-induced or -binding proteins, among them an indole-3-acetic acid amido synthetase and an auxin efflux transporter similar to AtPin5, were mostly downregulated in TR185 compared with WT. A transcript encoding a homeobox transcription factor similar to the transcription factor IFL of AtPIN1 was differentially expressed in response to G×N effect, with a lower expression in TR185 in response to LN only. Differential expression was also observed for various transcripts involved in the metabolism of cytokinin, with, in TR185 compared with WT, upexpression of a transcript involved in its degradation and downexpression of two transcripts possibly involved in its signalling. An upregulation of a gene encoding an ethylene-responsive transcription factor and a downregulation of transcripts involved in gibberellin synthesis or signalling were also observed in TR185. Lastly, two main genes involved in jasmonate metabolism were also differentially regulated between TR185 and WT, with downregulation of transcripts encoding the lipoxygenase AtLOX1 and upregulation of transcripts encoding the lipoxygenase AtLOX5.

**Table 4.** Differentially accumulated transcripts between TR185 and wild type, annotated as related to hormone metabolism and transportAverage Affymetrix GeneChip normalized expression values across three biological replicates for the mutant TR185 and the wild type in high and low N conditions. G, N, and G×N indicate transcripts responsive to genotype, nitrate, and genotype×nitrate effect, respectively (adjusted *P*-values <0.05). *G* and G indicate upregulation and downregulation, respectively, in TR185 compared with the wild type.

Target identifier	Annotation	MHN	MLN	WTHN	WTLN	Effect
Auxin metabolism						
Mtr.6663.1.s1_at	Indole-3-acetic acid amido synthetase	6.63	5.73	5.19	5.07	G
Mtr.45413.1.s1_at	Indole-3-acetic acid-amido synthetase	2.57	2.57	3.62	3.42	G
Mtr.14314.1.S1_at	Auxin-induced protein 5NG4	4.41	4.76	5.43	6.51	G
Mtr.39011.1.S1_at	Auxin-binding protein ABP19b precursor	8.04	8.60	9.26	9.49	G
Mtr.49764.1.s1_at	Auxin:hydrogen symporter similar to AtPin5	2.08	2.16	3.04	3.39	G
Mtr.49221.1.s1_at	Transcription factor similar to IFL	4.89	4.11	3.55	4.68	G×N
Mtr.11046.1.s1_at	Transcription factor similar to AtHB2	5.79	4.88	4.58	3.96	G
Cytokinin metabolism						
Mtr.11675.1.s1_at	Cytokinin dehydrogenase	6.01	6.91	5.25	5.62	G
Mtr.38123.1.s1_at	Transcription factor similar to APRR2	4.20	4.16	5.07	5.90	G
Mtr.8550.1.S1_s_at	Oxygen transporter activity	6.63	8.34	8.39	9.12	G N
Ethylene metabolism						
Mtr.21627.1.s1_at	Ethylene-responsive transcription factor	7.53	6.65	6.08	5.89	G
Gibberelin metabolism						
Mtr.6537.1.s1_s_at	Gibberellin 20-oxidase	6.73	7.25	7.87	8.01	G
Mtr.26011.1.s1_at	Gibberellin 20 oxidase 1-B	6.50	6.78	7.65	7.78	G
Mtr.7253.1.s1_at	Gibberellin-regulated family protein	6.11	6.25	7.95	7.61	G
Mtr.47463.1.s1_at	Scarecrow transcription factor family protein	3.24	3.77	4.28	4.76	G
Jasmonate metabolism						
Mtr.46868.1.s1_s_at	Lipoxygenase similar to AtLOX1	11.50	11.92	12.68	12.67	G
Mtr.46870.1.s1_at	Lipoxygenase similar to AtLOX1	10.33	11.03	11.60	11.84	G
Mtr.46864.1.s1_at	Lipoxygenase similar to AtLOX1	4.61	5.03	6.19	5.81	G
Mtr.50426.1.s1_at	Lipoxygenase similar to AtLOX1	4.69	5.47	6.07	5.77	G
Mtr.8427.1.s1_at	Lipoxygenase similar to AtLOX1	4.54	5.73	6.45	6.53	G
Mtr.20079.1.s1_at	Lipoxygenase similar to AtLOX1	2.88	2.99	3.20	4.36	G
Mtr.3795.1.s1_at	Lipoxygenase similar to AtLOX5	4.64	5.70	4.17	4.10	G
Mtr.8452.1.s1_at	Lipoxygenase similar to AtLOX5	5.60	4.73	4.34	3.97	G

## Discussion

The size and architecture of the root system determine the surface area of exchange between roots and the soil medium, and both are known to adapt in response to fluctuations of nutrient availability. Among the key nutrients, NO_3_^–^ is well known to markedly affect root system architecture. This work reports a new highly branched *M. truncatula* mutant, TR185, which lacks the capacity to adapt root architecture to nitrate supply and shows an unexpectedly low nitrogen acquisition. TR185 was selected among various gamma-ray mutants because of its highly branched root phenotype and expected enhanced nitrogen acquisition; its numerous young roots, which had not yet developed strong lignin barriers, were predicted to exploit more efficiently the soil for uptake of both water and nutrients (Steudle and Peterson, 1998; Naseer *et al.*, 2012). However, the current study demonstrated that TR185 displayed N-limited responses; under both LN and HN supply, TR185 was depressed in shoot and root dry weight and had a preferential dry weight allocation to roots at the expense of shoots compared with WT. The suboptimal N nutrition of TR185 became evident as the growth cycle progressed, as from the 21-day stage, TR185 had lower %ShootN than the WT at both nitrate conditions. Furthermore, its low NUR and high amount of leaf area produced per N acquired were both typical for plants under very low N status (Larigauderie *et al.*, 1994; Moreau *et al.*, 2012). In *Arabidopsis*, root N uptake and architecture are both known to be regulated by external N supply and internal N demand. Based on these well-known responses in *Arabidopsis*, the current work investigated whether TR185 is impaired in either local acquisition/perception of nitrate availability or in systemic regulation by nitrogen status of the whole plant.

Molecular studies in *Arabidopsis* have highlighted that nitrate *per se* is a signal leading to upregulation of N transporters and thus N acquisition in plants that have been previously N starved (Lejay *et al.*, 1999; Wang *et al.*, 2003; Scheible *et al.*, 2004; Bi *et al.*, 2007). The localized stimulatory effect of external nitrate on LR elongation and/or emergence has also been thoroughly investigated (Zhang and Forde, 1998; Remans *et al.*, 2006a; Krouk *et al.*, 2010; Gojon *et al.*, 2011). Nitrate has been demonstrated to be itself the signal for the stimulation of LR emergence (thus LR number) and elongation. This stimulation has been associated with an enhanced auxin accumulation in apex of root primordia in newly emerged LRs and has been shown to involve both ANR1 and NRT1.1 genes. In the current study, microarray analysis revealed an upregulation of various transcripts belonging to NO_3_^–^ or NH_4_^+^ transporters families in TR185 compared with WT. Such upregulation occurred irrespective of N supply and could thus indicate a permanent local perception of high nitrate availability in the mutant. However, this upregulation did not increase either root amino acid content or %RootN in TR185 compared with WT. Furthermore, no effect of nitrate supply on LR elongation or number was observed in TR185. Moreover, its highly branched root architecture is not representative of plants impaired in local perception of nitrate; its reduced responsiveness differed from that observed in *nrt1.1* mutants or *ANR1*-repressed lines of *Arabidopsis*, in which LR number was never higher than in the WT even under high nitrate availability. Taken together, these molecular and developmental responses of TR185 to N availability indicate that its highly branched root system architecture is not mainly induced by an impaired local perception of nitrate availability.

Besides the local stimulatory effect of nitrate, a feedback repression is known to be exerted by high N status of the whole plant, which downregulates high-affinity N transporters, whereas N starvation results in the opposite response. Specific members of the NRT2 and AMT1 families in *Arabidopsis* and MtNRT2 genes in *M. truncatula* are known to be involved in this response (Loque *et al.*, 2006; Yuan *et al.*, 2007; Ruffel *et al.*, 2008; Okamoto *et al.*, 2009; Girin *et al.*, 2010). A systemic repression of LR development by the high N status of the plant has also been described in *Arabidopsis*. A nitrate-dependent signalling pathway controlling LR elongation has been described, in which nitrate supply above 10mM blocks the elongation of LR post emergence in response to a high shoot nitrate accumulation (Zhang *et al.*, 1999; Zhang and Forde, 2000; Remans *et al.*, 2006b). More recently, an additional signalling pathway has been pointed out, in which LR emergence is controlled by N-assimilation products. According to Gifford *et al.* (2008), GLN is the predominant signal regulating repression of LR emergence, but an inhibition of root growth by ASN was also shown by Ivanov *et al.* (2012) and its possible role as an N-satiety signal suggested. In the current study, molecular and developmental analyses converge to indicate that the mutant could perceive a permanent N-starvation signal, which induced modification of root N acquisition and architecture compared with WT. Indeed, the upregulation of root N transporters in TR185 compared with WT could be characteristic of the N-starvation status in the mutant. Importantly, the decreased GLN and ASN root content in TR185 compared with WT could explain its highly branched root architecture in agreement with Gifford *et al.* (2008) and Ivanov *et al.* (2012). Grafting experiments revealed that the highly branched root phenotype in TR185 was transmissible from shoots and not from roots (Fig. 3), thus reinforcing the hypothesis of phloem transport of a signal in the mutant. As the GLN and ASN shoot contents were also lower in TR185 than in the WT, the signal could be the low GLN/ASN phloem content itself. The hypothesis does not exclude a possible higher degradation in roots of these two major N-storage forms, as suggested by the observed differential expression of transcripts involved in ammonium assimilation. As such, the lower root content of GLN and ASN could be related to the upregulation of a glutamate synthase and a putative l-asparagine amidohydrolase respectively in TR185 compared with WT.

Additional analyses of expression of genes involved in cell-wall modification, phenylpropanoid pathway, and hormone transport confirmed that TR185 plants were under N starvation and provided further explanation of their root architecture. Most of the genes involved in cell-wall degradation were upregulated in TR185 compared with WT, whereas those involved in cell-wall synthesis were downregulated. Such modifications have been previously observed for *Lotus japonicus* in N-starvation conditions (Omrane *et al.*, 2009). Most of the transcripts encoding cell-wall arabinogalactan proteins were downregulated in TR185, in agreement with the reduced root elongation observed in *Arabidopsis* AGP-defective mutants (van Hengel and Roberts, 2002; Shi *et al.*, 2003; Seifert and Roberts, 2007). Widespread differential expression of transcripts for the phenylpropanoid pathway was also observed between the two genotypes. The phenylpropanoid pathway serves as a rich source of metabolites in plants, being required for the biosynthesis of both lignin and many other important compounds such as the flavonoids (Fraser and Chapple, 2011). A large number of genes involved in lignin synthesis were repressed in TR185, whereas transcripts encoding a chalcone synthase or involved in flavonol glycoside synthesis were upregulated. The slightly lower root lignin content observed in TR185 compared with WT was in agreement with these results (Supplementary Fig. S5 available at *JXB* online). The higher expression in TR185 of genes involved in flavonoid synthesis support the hypothesis that the mutant was under permanent N starvation, in agreement with previous observations in *L. japonicus* roots (Omrane *et al.*, 2009). Flavonoid accumulation is known to decrease polar auxin transport, inducing a deregulation of LR elongation and thus short root architecture (Peer *et al.*, 2004; Peer and Murphy, 2007; Laffont *et al.*, 2010). Interestingly, most of the transcripts encoding auxin-induced or -binding proteins or related to the auxin efflux transporters PIN were downregulated in TR185 compared with WT, suggesting a decreased root auxin accumulation or transport. Differentially expressed genes between TR185 and WT related to other hormones could also explain TR185 root architecture, in particular the upregulation of a transcript involved in cytokinin degradation and, thus, enhanced LR initiation (Laplaze *et al.*, 2007) and the downregulation of transcripts involved in GA synthesis, which depressed root elongation (Beemster and Baskin, 2000; Achard *et al.*, 2003; Benkova and Hejatko, 2009).

In conclusion, the mutant TR185 displayed highly branched root architecture and impaired N acquisition, both irrespective of nitrate supply. Physiological and developmental analyses of its responses to N supply suggested that the root architecture of TR185 results from a systemic regulation by the plant nitrogen status, possibly involving GLN or ASN signals. Altered expression of genes of the phenylpropanoid pathway could also explain its root architecture. Further studies are needed both to determine in which gene the mutation occurred and fully understand the TR185 phenotype under conditions when it is relying exclusively on symbiotic N fixation for its N acquisition. Such results will identify the genes and physiological mechanisms that regulate legume root architecture and activity as a function of plant N status and give new targets for legume breeding.

## Supplementary material

Supplementary data are available at *JXB* online.

Supplementary Fig. S1. Quantitative reverse-transcription PCR validation of differentially accumulated transcripts initially identified by Affymetrix GeneChip analysis.

Supplementary Fig. S2. Relationship between shoot N concentration and shoot dry weight.

Supplementary Fig. S3. Contents of 10 amino acids in wild-type and mutant shoots under high and low nitrate supply at 14 and 21 days after germination.

Supplementary Fig. S4. Contents of eight amino acids in wild-type and mutant roots under high and low nitrate supply at 14 and 21 days after germination.

Supplementary Fig. S5. Root lignin content of the wild type and the mutant under high and low nitrate supply at 14 and 21 days after germination.

Supplementary Table S1. Genetic analysis of the highly branched root mutant TR185.

Supplementary Table S2. Primers used for quantitative reverse-transcription PCR.

Supplementary Table S3. The 75 differentially accumulated transcripts responsive to N supply and common to those identified by Ruffel *et al.* (2008) as responsive to either local nitrate starvation or systemic signals.

Supplementary Data
