# Supplementary material for: Unexpectedly low nitrogen acquisition and absence of root architecture adaptation to nitrate supply in a Medicago truncatula highly branched root mutant
Source: J Exp Bot. 2014 Apr 4;65(9):2365–80. doi: 10.1093/jxb/eru124 (PMC4036509; doi:10.1093/jxb/eru124)
Supplement: Supplementary Data [file supp_65_9_2365__index.html]

Unexpectedly low nitrogen acquisition and absence of root architecture adaptation to nitrate supply in a Medicago truncatula highly branched root mutant — Unexpectedly low nitrogen acquisition and absence of root architecture adaptation to nitrate supply in a Medicago truncatula highly branched root mutant — Supplementary Data 

# Unexpectedly low nitrogen acquisition and absence of root architecture adaptation to nitrate supply in a *Medicago truncatula* highly branched root mutant

## Supplementary Data

Data files

**Files in this Data Supplement:**

- Supplementary Data - Supplementary Data
