# Supplementary material for: Unexpectedly low nitrogen acquisition and absence of root architecture adaptation to nitrate supply in a Medicago truncatula highly branched root mutant
Source: J Exp Bot. 2014 Apr 4;65(9):2365–80. doi: 10.1093/jxb/eru124 (PMC4036509; doi:10.1093/jxb/eru124)
Supplement: Supplementary Data [file supp_eru124_jexbot106021_file001.pdf]

Unexpectedly low nitrogen acquisition and absence of root architecture adaptation to nitrate supply in a *Medicago truncatula* highly-branched root mutant

Virginie Bourion[1]*. Chantal Martin[1]. Henri de Larambergue[1]. Françoise Jacquin[1]. Grégoire Aubert[1]. Marie-Laure Martin-Magniette[23456]. Sandrine Balzergue[456]. Geoffroy Lescure[7]. Sylvie Citerne[7]. Marc Lepetit[8]. Nathalie Munier-Jolain[1]. Christophe Salon[1]. Gérard Duc[1]

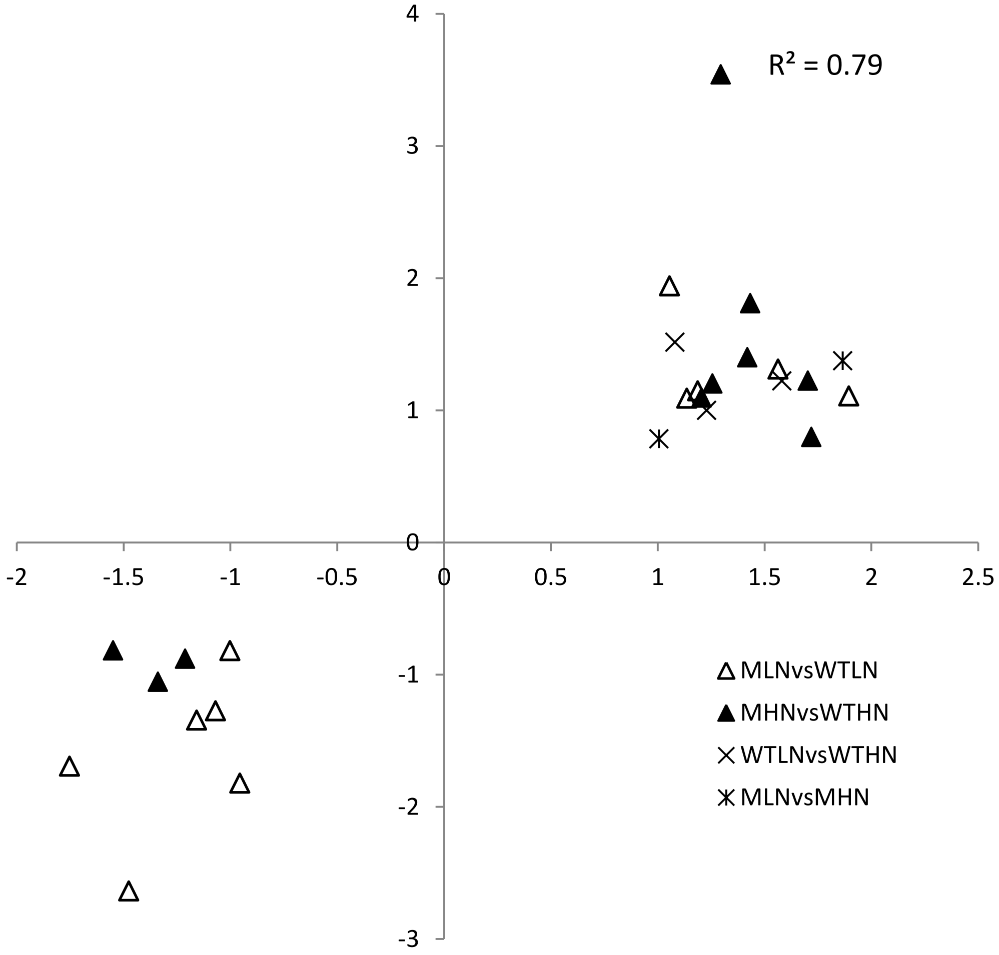

Supplementary Figure 1. Analysis by Q-RT-PCR of the accumulation of differentially accumulated transcripts. initially identified by Affymetrix GeneChip analysis. on the three biological replicates.

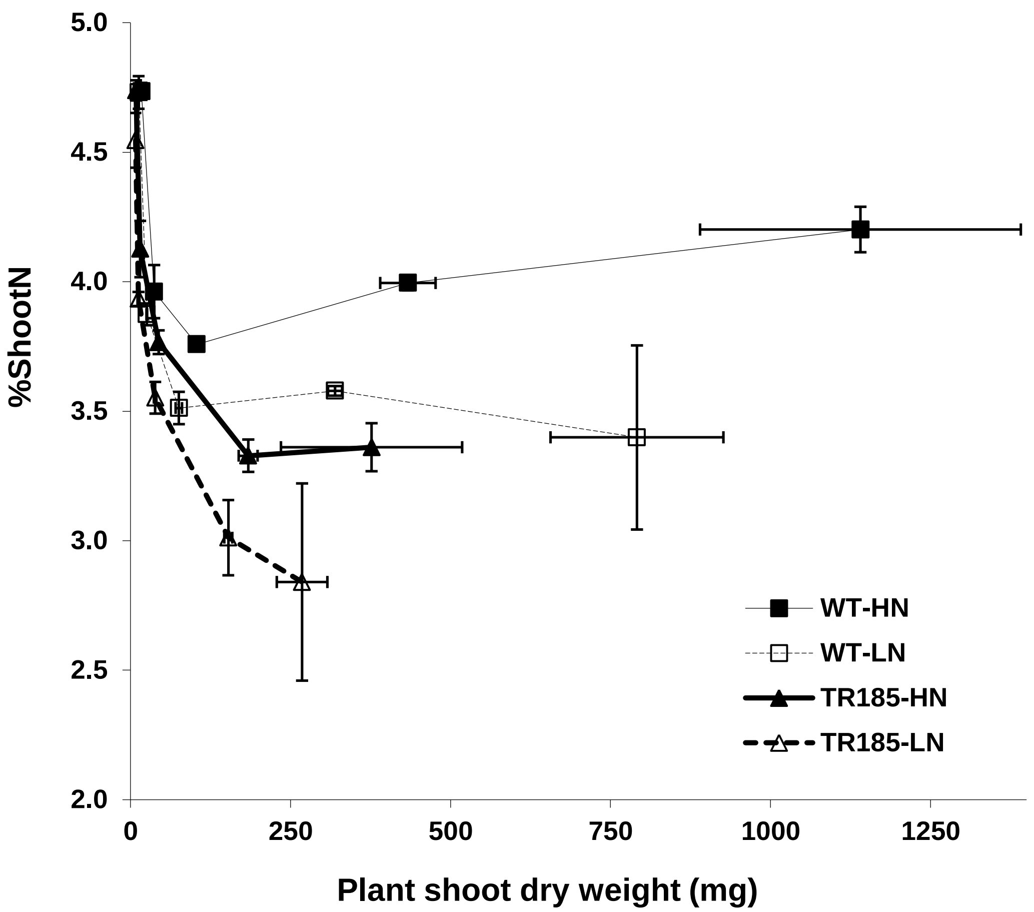

Supplementary Figure 2. Relationship between shoot N concentration (%ShootN) and shoot dry weight (SDW). At each date. data are means from three biological replicates of six plants each ± SE.

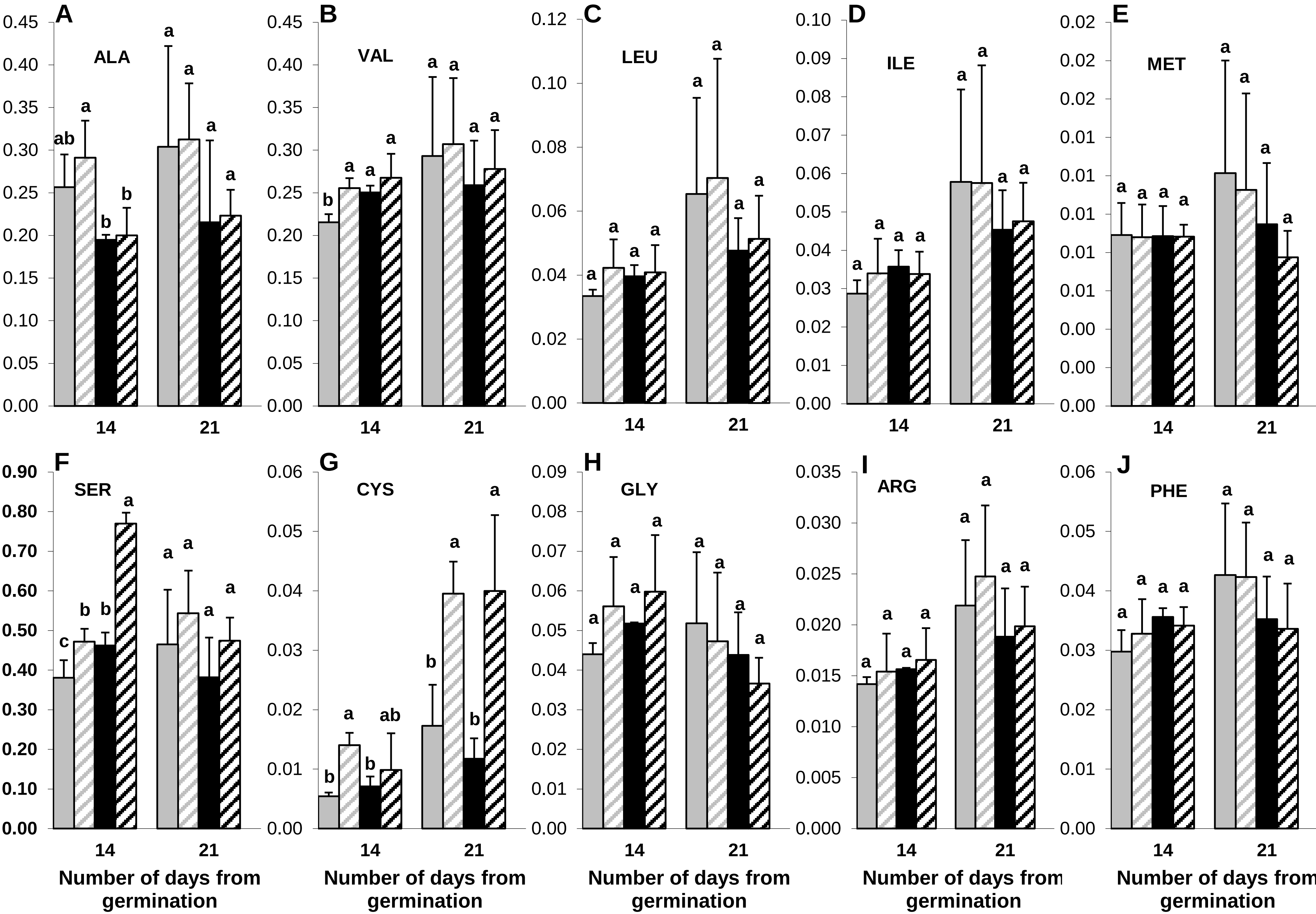

Supplementary Figure 3: Shoot content of ten amino acids

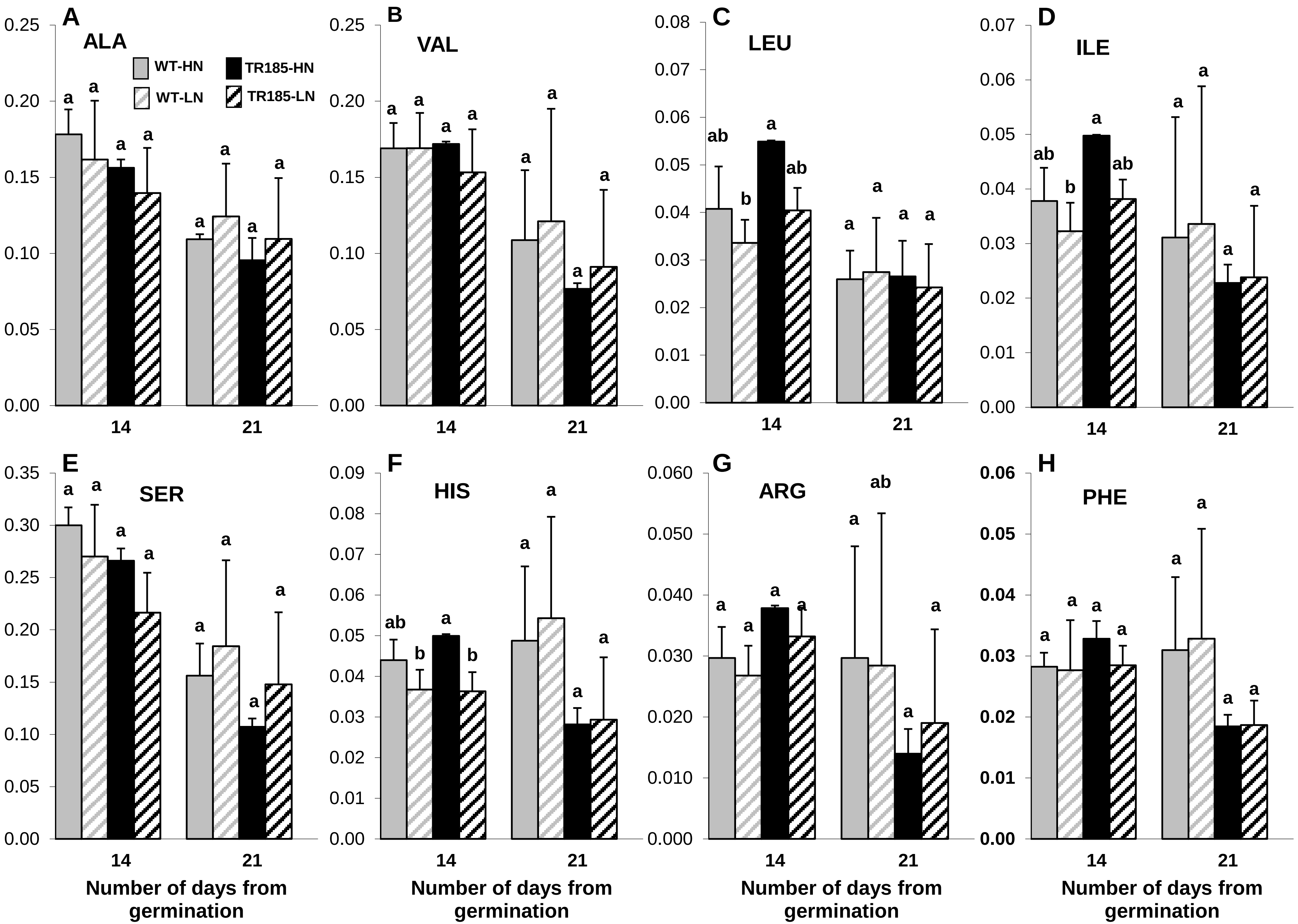

Supplementary Figure 4: Root content of eight amino acids

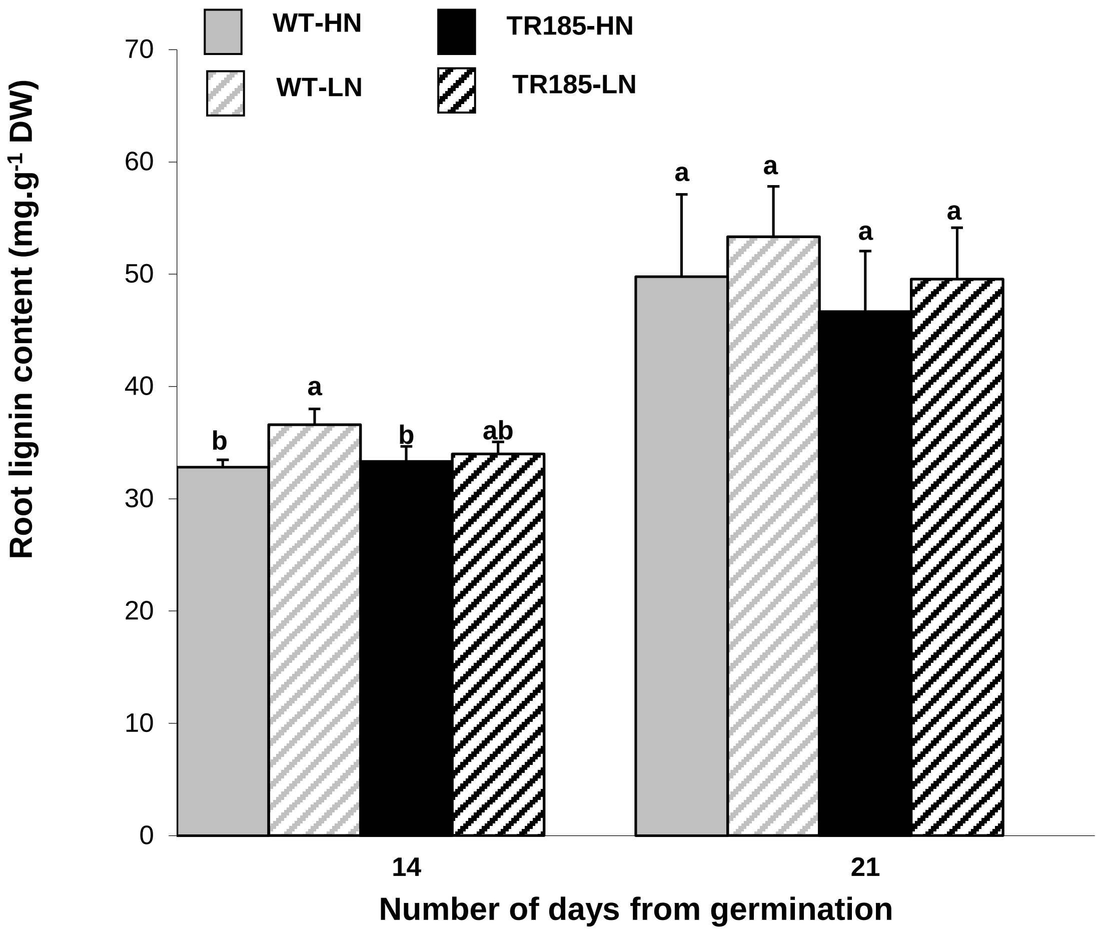

Supplementary Figure 5. Root lignin content of the wild-type (WT) and the mutant (TR185) under high (10 mM. [HN]) or low (1 mM. [LN]) nitrate supply at 14 and 21 days after germination. At each date. data are means from three biological replicates of six plants each ± SE and means followed by different letters are significantly different based on multiple comparisons (LSD test) at $p < 0.05$.

Supplementary Table 1. Genetic analyses of the high-branched mutant TR185

Generation of ♀ x ♂ cross	High-branched root phenotype[a]	Wild-type root phenotype[a]	χ^2 value for 1:3 ratio
BC1 (TR185 x J5)	0	7	
BC1 (J5 x TR185)	0	5	
BC1S1 (TR185 x J5)	6	28	0.98
BC1S1 (J5 x TR185)	28	75	0.26

[a] Number of plants

Supplementary Table 2. Primers used for Real Time RT-Q-PCR assays

Target Id[a]	primer sequence (5' -> 3')
Mtr.10626.1.S1_at	TGCGACGAAGATAAAGGAATGTGG
	GAACTGGGGGATTAACACAAAAGG
Mtr.14314.1.S1_at	GGGCAAAAAGCAAAGAAGGAATCAC
	TACATATATTTTGGCTCCAGAGGAG
Mtr.16885.1.S1_at	GATCATGAAGCATCTTTCCTGAGAG
	GGCAGATTACAAAGCTCAAAATTCG
Mtr.18380.1.S1_at	TATGGAAAGTGGATGTCAGTTGGAC
	GTAAAAGAACGTGCGTGTTCTGAAG
Mtr.20107.1.S1_at	AAGCTGGGTCTGTTTATGACTCAC
	AGCACTAGAGATAACATTCTTGGAC
Mtr.20354.1.S1_at	CTTGATTCAGTTGATTGATGAGCTG
	GGTGTTTCATCATGCTACACCTCC
Mtr.22383.1.S1_x_at	GAAGAGTCTCCAAGTGGATGTG
	ATGAATCAGTACCATTCCAAATCCC
Mtr.23663.1.S1_at	ACACCACCGTGCTCTGTTTCCTC
	TTGGATAGTGAGTGGAGGTGAAGC
Mtr.25576.1.S1_at	TACATAACCCTCTCGATCGATTTCC
	TGATGGAGAGAAACTTAGTCTCTAC
Mtr.26011.1.S1_at	GAGGAAGGTTTGAAAAAGTTGGTTTC
	TGAACAGATTTTCACTTACGCAACC
Mtr.37034.1.S1_at	CTCTCTACTTGATCAATCGTCTTGG
	CTAAGTTATGTCAACATAGTCAACG
Mtr.4076.1.S1_at	ATGGAAGCACCTCTTCGTTGAAGC
	ACTACCAAATCCATACGACCGTATC
Mtr.40997.1.S1_s_at	GCGGCTTTCTTGATTGCCTCTAC
	CTGGATGTAGAAATAGAAGAAGGTG
Mtr.41610.1.S1_at	GCACTCACACTCCTCTCATTCC
	TAGGGTTTGGACAGTCAGAAACTC
Mtr.43680.1.S1_at	CCGAACACATTGCCTTTCAAGTGC
	GGGGTAAAAGGATTGGTGCCAG
Mtr.43830.1.S1_at	CAGTTTTCACTTCATTTGCTTTGCC
	CCCAATGAATTAGACCAAACTAGAG
Mtr.51607.1.S1_at	GGAAAAGAATGGAAGCACCTCTTCG
	ACTACCAAATCCATACGACCGTATC
Mtr.8452.1.S1_at	CTGTGAAGATGCCATATACACTGC
	CAACAACACAACAACGACGATGAC
EF1B	GGTTGAGGATCGTCTTACTGCTG
	AATGTCTGCCCACTACCATGATC
Ubiquitin	CCAGAAGGAATCCACTCTTCA
	CTTCCCACAATAATGACGATC

[a] Target identifier (Affymetrix) or reference genes for Q-RT-PCR

Supplementary Table 3. The 75 differentially accumulated transcripts responsive to N effect and common to those identified by Ruffel et al. (2008) as responsive to either local nitrate starvation (LNO3vs-NO3) or systemic signals (LNO3vsSNO3)

Affymetrix probe	WTLNvsWTHN[a]	MLNvsMHN[a]	LNvsHN[a]	MXLN[a]	LNO3vs-NO3[b]	LNO3vsSNO3[b]	Annotation Ruffel
Mtr.24492.1.S1_s_at	1.57	-	1.25	-	-37.27	-	NAD(P)H-quinone oxidoreductase chain H. chloroplast
Mtr.40997.1.S1_s_at	-	1.87	1.55	-	-29.04	-	Photosystem I P700 chlorophyll A apoprotein A1
Mtr.14625.1.S1_at	1.44	-	1.14	-	-18.77	-	Photosystem I assembly Ycf4
Mtr.14636.1.S1_s_at	-	-	1.11	-	-18.77	-	RNA polymerase beta subunit Rpb2-like
Mtr.45608.1.S1_s_at	1.74	-	0.99	-	-18.00	-	Unknown
Mtr.45626.1.S1_s_at	1.60	-	1.21	-	-15.67	-	GAP|atpH
Mtr.49039.1.S1_s_at	-	1.60	0.99	-	-15.35	-	H+-transporting two-sector ATPase. alpha/beta subunit
Mtr.14042.1.S1_s_at	-	1.46	-	-	-12.47	-	GAP|petD
Msa.1055.1.S1_at	1.69	-	1.08	-	-12.13	-1.07	NADH-ubiquinone oxidoreductase chain
Mtr.45639.1.S1_s_at	1.40	-	1.09	-	-12.13	-	GAP|atpB
Mtr.14644.1.S1_s_at	-	-	0.96	-	-11.24	-	Photosystem I psaA and psaB
Msa.3141.1.S1_at	-	1.94	1.21	-	-11.08	-	ATP synthase subunit alpha
Mtr.14061.1.S1_s_at	-	-	0.95	-	-9.85	-	GAP|psaA
Mtr.45615.1.S1_s_at	-	-	1.03	-	-9.19	-	from 54343 to 54705
Mtr.31123.1.S1_at	1.38	-	0.98	-	-8.69	-	similar to UP|Q9SYP1 (Q9SYP1) F9H16.5 protein
Mtr.18232.1.S1_s_at	1.51	-	1.16	-	-8.34	-	AAA ATPase; H+-transporting two-sector ATPase
Msa.2071.1.S1_s_at	-	1.80	1.18	-	-8.17	-1.09	Unknown
Mtr.45637.1.S1_s_at	-	-	1.23	-	-7.46	-	GAP|ndhK
Mtr.36986.1.S1_s_at	-	-	0.97	-	-6.92	-	NADH dehydrogenase 19kDa subunit
Mtr.29056.1.S1_at	-	-	0.95	-	-6.28	-	Unknown
Mtr.37266.1.S1_s_at	-	2.86	1.85	-2.02	-5.21	-	Unknown
Mtr.10097.1.S1_at	-	-	0.98	-	-5.17	-	Unknown
Mtr.44795.1.S1_at	-	-	1.10	-	-5.06	-	similar to UP|Q76JT3 (Q76JT3) RelA-SpoT like protein PsRSH1
Mtr.43508.1.S1_at	1.86	-	0.98	-	-4.72	1.82	Auxin-induced protein
Mtr.31694.1.S1_at	1.44	-	-	-	-4.72	-	similar to UP|Q84KJ4 (Q84KJ4) Myosin XI (Fragment)
Mtr.29652.1.S1_at	1.96	-	1.19	-	-4.66	-	Unknown
Mtr.14053.1.S1_s_at	-	1.51	-	-	-4.26	-	GAP|atpF
Mtr.33189.1.S1_s_at	-	-	1.13	-	-3.94	-	AML1
Mtr.34841.1.S1_s_at	-	1.57	1.14	-	-3.76	-	CP43 chlorophyll apoprotein of photosystem II
Mtr.33045.1.S1_at	-	-	0.96	-	-3.41	-	similar to UP|Q94KD1 (Q94KD1) At1g05960 T21E18_20
Msa.1917.1.S1_at	-	1.66	0.99	-	-3.01	-	Unknown
Mtr.44175.1.S1_at	-	-	0.95	-	-3.01	-	similar to UP|Q8RY22 (Q8RY22) AT3g03380/T21P5_20
Mtr.38167.1.S1_at	1.41	-	-	-	-2.85	-	Cationic peroxidase 1 precursor
Mtr.39306.1.S1_at	1.40	-	1.26	-	-2.79	-	Sob protein
Mtr.33172.1.S1_at	-	-	1.04	-	-2.62	-	weakly similar to UP|Q5ZCB9 (Q5ZCB9) Ubiquitin-conjugating enzyme
Mtr.44875.1.S1_at	1.37	-	-	-	-2.45	-	weakly similar to UP|Q6K6B1 (Q6K6B1) CLIP-associating protein
Mtr.44882.1.S1_at	-	1.47	1.08	-	-2.36	-	Unknown
Mtr.29714.1.S1_at	-	1.34	-	-	-2.31	-	Unknown

[a] Only significant differential expressions are shown. in a log2 scale.
[b] Fold-change of transcript accumulation. as indicated by Ruffel et al. (2008)

Supplementary Table 3. End

Affymetrix probe	WTLNvsWTHN[a]	MLNvsMHN[a]	LNvsHN[a]	MXLN[a]	LNO3vs-NO3[b]	LNO3vsSNO3[b]	Annotation Ruffel
Mtr.14599.1.S1_x_at	1.37	-	-	-	-2.16	-	LQGC hypothetical protein
Mtr.28638.1.S1_at	-	-	0.97	-	-2.04	-	Unknown
Mtr.13860.1.S1_at	-	-	0.97	-	-2.04	-	weakly similar to UP|WIS1_SCHPO (P33886) Protein kinase wis1 (sty2)
Mtr.33744.1.S1_s_at	1.34	-	-	-	-1.92	-	similar to UP|Q6AVJ1 (Q6AVJ1) Expressed protein
Mtr.13713.1.S1_at	-	-	1.02	-	-1.89	-	Unknown
Mtr.4087.1.S1_at	1.54	-	0.96	-	-1.83	-	OMNI|NTL01LJ12 ABC transporter ATPase component
Mtr.35635.1.S1_at	1.49	-	-	1.92	-1.79	-	Unknown
Mtr.38820.1.S1_at	-	-	0.97	-	-1.68	-	cytochrome P450
Mtr.1527.1.S1_at	1.37	-	-	-	-1.65	-	Unknown
Mtr.5936.1.S1_at	-	-	0.95	-	-1.58	-	Unknown
Mtr.10025.1.S1_at	1.71	-	0.98	-	-1.53	-	Phosphoribosylanthranilate transferase-like protein
Mtr.5823.1.S1_a_at	-	-	0.99	-	-1.49	-	similar to UP|Q7Y0H3 (Q7Y0H3) CLV1-like receptor kinase
Mtr.6021.1.S1_at	1.37	-	-	-	-1.48	-	Unknown
Mtr.32291.1.S1_at	1.72	-	1.00	-	-1.47	-	similar to PIR|JC4209|JC4209 GTP cyclohydrolase II
Mtr.6373.1.S1_at	-	-	0.99	-	-1.37	-	weakly similar to UP|Q40326 (Q40326) Acetyl-CoA carboxylase
Mtr.39559.1.S1_at	1.64	-	-	1.96	-1.33	-	Unknown
Mtr.8799.1.S1_at	1.56	-	-	-	-1.32	-1.41	Raffinose synthase
Mtr.32561.1.S1_at	-	-	1.06	-	-1.32	-	weakly similar to UP|Q9FHH2 (Q9FHH2) 101 kDa heat shock protein
Mtr.9569.1.S1_at	-	1.40	-	-	3.05	1.71	Thaumatin-like protein 1
Mtr.39456.1.S1_at	1.63	1.58	1.61	-	3.10	-	weakly similar to Q9LW87 Coatomer protein complex
Mtr.8550.1.S1_s_at	-	1.71	1.22	-	127.12	9.06	Leghemoglobin
Mtr.8284.1.S1_s_at	-	1.82	1.32	-	196.72	-	Leghemoglobin
Mtr.10382.1.S1_at	1.91	-	0.98	-	-	32.56	B12D-like protein
Mtr.4531.1.S1_s_at	1.83	-	1.03	-	-	30.48	B12D-like protein
Mtr.8645.1.S1_at	1.57	-	1.20	-	-	-1.06	Riboflavin biosynthesis protein ribA. chloroplast precursor
Mtr.48109.1.S1_at	1.36	-	-	-	-	-1.25	Putative WRKY4 transcription factor
Mtr.25557.1.S1_at	-	1.39	1.13	-	-	-1.34	1-aminocyclopropane-1-carboxylate oxidase
Mtr.31237.1.S1_at	-	-	0.98	-	-	1.09	Hydrolase-like protein
Mtr.48070.1.S1_at	-	-	0.95	-	-	1.24	Unknown
Mtr.42876.1.S1_at	-	1.38	-	-	-	-1.15	Pathogenesis-related protein 4A
Mtr.23567.1.S1_x_at	-	1.36	-	-	-	-1.26	Unknown
Mtr.7380.1.S1_at	-1.42	-	-	-	-	1.69	Unknown
Msa.905.1.S1_at	-1.67	-	-	-	9.51	3.73	Leghemoglobin
Mtr.29429.1.S1_at	-	-1.41	-	2.12	-	-1.12	Unknown
Mtr.49372.1.S1_at	-	-2.30	-	3.19	-1.48	-	Unknown
Mtr.12474.1.S1_at	-	-	-1.02	-	-	-1.13	Isoflavonoid glucosyltransferase
Mtr.43282.1.S1_at	-1.37	-	-1.15	-	2.60	-5.48	S-adenosyl-L-methionine: 2.7.4'-trihydroxyisoflavanone 4'-O-methyltransferase

[a] Only significant differential expressions are shown. in a log2 scale.
[b] Fold-change of transcript accumulation. as indicated by Ruffel et al. (2008)
